# Nutritional Knowledge about Maternal and Newborn Health among Physiotherapists during the COVID-19 Pandemic in Minas Gerais, Brazil

**DOI:** 10.3390/nu16020180

**Published:** 2024-01-05

**Authors:** Isabelle Marinho, Maria-Raquel G. Silva, Teresa Paiva, Rita Santos-Rocha, Hugo-Henrique Silva

**Affiliations:** 1 School of Health Sciences, University Fernando Pessoa, 4249-004 Porto, Portugal; 2 FP-I3ID, FP-BHS, CEBIMED and Faculty of Health Sciences, University Fernando Pessoa, 4200-150 Porto, Portugal; 3 CIAS—Research Centre for Anthropology and Health—Human Biology, Health and Society, University of Coimbra, 3000-456 Coimbra, Portugal; 4 CHRC—Comprehensive Health Research Centre, Nova Medical School, Nova University of Lisbon, 1150-090 Lisbon, Portugal; teresapaiva0@gmail.com; 5 Gymnastics Federation of Portugal—Scientific Committee, 1600-159 Lisbon, Portugal; 6 CENC—Sleep Medicine Centre, 1649-035 Lisbon, Portugal; 7 ESDRM-IPSANTARÉM Sport Sciences School of Rio Maior, Polytechnic Institute of Santarém, 2001-904 Rio Maior, Portugal; ritasantosrocha@esdrm.ipsantarem.pt; 8 CIPER—Laboratory of Biomechanics and Functional Morphology, Interdisciplinary Centre for the Study of Human Performance, Faculty of Human Kinetics, University of Lisbon, 1499-002 Lisbon, Portugal; 9 ICBAS—School of Medicine and Biomedical Sciences, University of Porto, 4050-313 Porto, Portugal; up200203100@edu.icbas.up.pt; 10 Portuguese Ministry of Education, 1399-025 Lisbon, Portugal; 11 Leixões Sport Clube, Senior Female Volleyball Team, 4450-277 Matosinhos, Portugal

**Keywords:** maternal health, newborn health, pregnancy, nutrition, nutritional knowledge, physiotherapist

## Abstract

Adequate nutrition before and during pregnancy, as well as postpartum, is among the major contributors to maternal and newborn health. Physiotherapists’ knowledge of this area is still scarce, although their clinical practice has been linked to newborns’ neuropsychomotor development, which, in turn, is influenced by maternal health and nutritional status. Therefore, this study aimed to evaluate the nutritional knowledge of physiotherapists regarding maternal and newborn health. A total of 70 Brazilian physiotherapists (32.2 ± 6.0 years; 72.9% females) were evaluated between November 2019 and February 2020 for their sociodemographic characteristics, professional experience, and nutritional knowledge about maternal and newborn health through a validated questionnaire personally administered by the same trained researcher. Most of the physiotherapists had graduated but had no specialization in maternal and child physiotherapy (96.1% of the females and all the males). The nutritional knowledge about maternal and newborn health was significantly different between the female and male health professionals, as well as between the less and more experienced participants, i.e., female physiotherapists and the more experienced ones had more correct answers on the nutritional questionnaire than the male and less experienced physiotherapists, respectively (*p* < 0.05). Our results open an interesting window for the future education and training of Brazilian physiotherapists in nutrition.

## 1. Introduction

**Figure 1:**
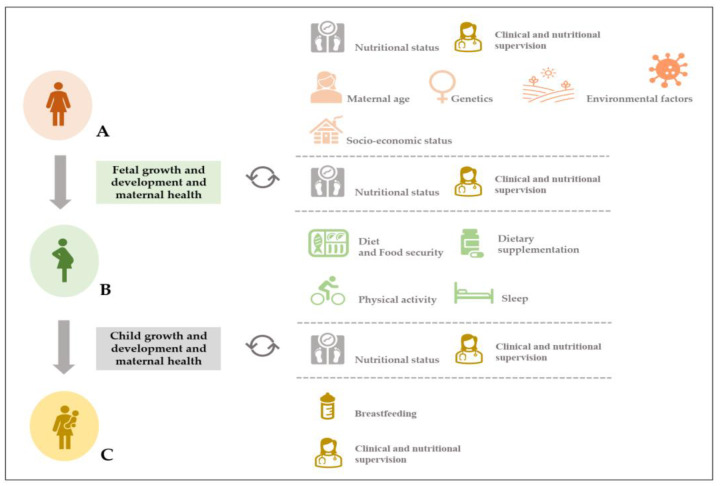
General framework related (but not limited) to women’s nutritional status before (pink images) and during (green images) pregnancy ((**A**) and (**B**), respectively) and postpartum (yellow images) (**C**), and other potential variables influencing fetal and child growth and development, respectively (adapted from Silva and Rodríguez, 2022) [5]. An appropriate nutritional status, which is associated with frequent clinical and nutrition supervision, is crucial for the maintenance and the promotion of good health in three important areas of women’s health and well-being (**A**–**C**). The maternal age, genetic profile, socioeconomic status, and environmental variables (including the influence of the pandemic caused by COVID-19) may affect maternal health and fetal growth and development (**A**). In addition, diet and food security, dietary supplementation, as well as the maintenance of an active lifestyle and good sleep hygiene can also be critical during pregnancy and for the child’s growth and development (**B**). To respond to the woman’s energy and nutritional needs, as well as those of the newborn (**C**), clinical and nutritional supervision are required to assess the maternal and newborn’s health and well-being.

Appropriate nutrition is a key element of both lifestyle and preventative medicine. Inadequate nutritional status is one of the most common risk factors for long-term health problems, and has been linked to the major causes of non-communicable diseases, such as obesity, cardiovascular diseases, diabetes, and cancers [1,2], which have been overall associated with premature death in 80% of cases [1]. Furthermore, it is well known that a poor nutritional status during pregnancy (e.g., gestational weight gain) can lead to a high risk of metabolic and cardiovascular diseases for the mother and her child in later life [3,4,5]. As a result, there is an urgent need for stronger preventive programs addressing nutritional and dietary risk factors during pregnancy and postpartum [6,7]. To mitigate these risks, the Institute of Medicine (IOM: now the National Academy of Medicine) has released specific guidelines for optimal health and nutrition [8] and recommends that health professionals should counsel women on the most adequate methods for body weight control before and during pregnancy, as well as provide dietary, physical exercise, and sleep hygiene advice [9], as shown in Figure 1.

Overweight and obesity are major public health problems [10]. Their incidence in pregnant women has been associated with newborn prematurity, neonatal infections, and cardiovascular, respiratory, and neurological disorders [7,9,11,12,13], with a high prevalence in low- and middle-income countries [14]. Promoting nutritional education in pre- and postnatal care routines can prevent neurological disorders in newborns [7,9,15], and future compromised functions in children’s static and dynamic balance, appendicular coordination, sensitivity, and motor coordination [16,17].

Since 1989, the World Health Organization [18] has worked to improve the dietary intake of pregnant women, but marginal intervention coverage and adherence have been observed [19,20].

Therefore, there is a need to support health professionals’ knowledge about the adequate nutritional profile of a pregnant woman and its legacy for the newborn’s health and neuromotor development. Although many countries have provided general guidance [21,22,23,24,25], the existing evidence suggests conflicting perspectives among women and health professionals about prenatal care, nutritional recommendations, and physical activity during pregnancy [3]. Health professionals like physiotherapists may consider nutrition education a low priority [3], but the importance of the physiotherapeutic action in promoting children’s health from pregnancy to the end of early childhood is crucial [26], and is not restricted to the treatment of diseases and rehabilitation [27]. Consequently, it is of the utmost importance that health professionals be educated on advising women on risk reduction and body weight management through nutrition education.

To properly educate pregnant women, physiotherapists should possess nutritional knowledge regarding the following aspects and recommendations. To support the mother’s metabolism changes, blood volume and red cell enlargement, and the transfer of energy and nutrients to the fetus, her energy and nutritional requirements are raised [5,7]. A balanced dietary intake should include sufficient energy, as well as vital micronutrients, such as iron, folate, zinc, calcium, vitamin D, and essential fatty acids, to promote red blood cell synthesis, bone development, enzyme activity, and brain development [28]. Although the recommended dietary allowances for vitamins D, E, and K, biotin, sodium, potassium, chloride, calcium, phosphorus, and fluoride do not increase during pregnancy, the estimated energy, protein, specific vitamins (vitamin A, thiamin, niacin, pantothenic acid, vitamin B-6, folate, vitamin B-12, and vitamin C) and minerals (choline, magnesium, iron, zinc, selenium, iodine, copper, manganese, chromium, and molybdenum) requirements are increased during the second and the third trimesters [29,30,31,32,33,34]. The consumption of fruits and vegetables, lean meats, low-fat dairy, and whole grains can promote the appropriate mother and newborn weight and, hence, a successful partum and postpartum period [7]. There are still no effective supplements that can be used to avoid unfavorable perinatal outcomes when there is an insufficient maternal nutritional intake, whether for a short or long term. Furthermore, supplements should only be used when a deficiency exists [5,7]. Special groups of pregnant women who do not normally follow a healthy diet, such as heavy cigarette smokers, heavy alcohol consumers, drug abusers, those carrying more than one fetus, and athletes, should begin taking a multivitamin–mineral preparation on a daily basis starting in the second trimester [7]. In spite of the great variety of infant formulas, breast milk is still the best option for a baby’s needs in terms of energy and nutrients, especially for those who are less than 12 months of age [5].

In addition to optimal nutrition, a healthy lifestyle during pregnancy should also involve daily physical exercise of moderate intensity and adequate sleep hygiene [9]. Evidence has suggested that increasing education and labor force participation among women might be relevant for addressing overweight/obesity in Latin America [35]. To better ensure physiotherapists’ positive interactions with pregnant women and to improve the adherence to clinical guidelines through training and practice standardization, there is a need to understand the relationship between current practices and the physiotherapists’ characteristics, as the latter might influence their practices. Indeed, evidence about the impact of nutritional knowledge of health professionals on maternal and newborn health, and underlying assumptions is still scarce. However, one of the few studies observed that only 38% physiotherapists had professional experience with pregnant women or women in postpartum (*p* < 0.01), and that being a female physiotherapist (*p* < 0.01) were substantially related with providing health education in maternal and newborn health [15]. These findings confirm our experience in maternal and child physiotherapy, which is dominated by female professionals, who improve their knowledge with additional graduated studies considering ongoing professional needs. Moreover, we have observed that Brazilian professionals lack nutritional education in academic curricula, since not all Brazilian institutions, responsible for teaching physiotherapy courses, integrate nutrition as a discipline in the context of maternal and newborn health. Therefore, this study aimed to evaluate the nutritional knowledge of physiotherapists about maternal and newborn health according to sex and professional experience.

## 2. Materials and Methods

### 2.1. Study Sample and Recruitment

This cross-sectional study was conducted between November 2019 and February 2020 in the city of Juiz de Fora, in the state of Minas Gerais—Brazil.

The participants were selected considering the following inclusion criteria: (1) being, at least, graduates in physiotherapy from a Brazilian university; (2) having at least one year of experience as a physiotherapist in Brazil; and (3) working in the city of Juiz de Fora, Minas Gerais (Brazil). Individuals with incomplete information in the questionnaire were excluded from the study.

A total of 153 physiotherapists were recruited and requested to complete an in-person questionnaire distributed in paper. The sample size was calculated using the online Epi Info sample size calculator supported by the Division of Health Informatics and Surveillance, and the Center for Surveillance, Epidemiology and Laboratory services [36]. The city of Juiz de Fora has 2102 physiotherapists registered in the Brazilian General Council for Physiotherapists [37]. A population proportion of 50% with an 80% confidence interval, assuming a type I error of 5%, was estimated, i.e., 153 physiotherapists from the city of Juiz de Fora. However, the final sample included 70 physiotherapists, who gave complete information in the questionnaire, representing 20% of the population proportion, with an 70% confidence interval and a margin of error of 5%.

This was a non-probabilistic convenience sample and health professionals were contacted through personal contacts in health centers and clinics. Due to the COVID-19 pandemic restrictions, data collection was interrupted, and the response rate was 95.9% after the validation of the survey. Additional characteristics of participants are described in Table 1.

The study was approved by the Ethical Committee of University Fernando Pessoa (Porto, Portugal, Reference: CEUFP10092018). All participants provided informed consent to participate in this study.

### 2.2. Study Procedures

Data regarding sociodemographic characteristics (age, sex, and type of work facility), professional experience as a physiotherapist (including if any nutrition subject was within academic curricula), and nutrition-related knowledge on maternal and newborn health were collected using a questionnaire personally administered to each participant by the same trained researcher.

#### 2.2.1. Professional Experience

Professional experience was assessed through educational level (bachelor, master or doctorate), professional experience as physiotherapist (less than 5 years, 5 to 10 years, 10 to 15 years, and more than 15 years), type of specialization level (postgraduate or specialization course), training related to maternal and child health, and if there was any nutrition subjects within the academic curricula.

#### 2.2.2. Nutrition-Related Knowledge on the Maternal and Newborn Health

The nutrition-related knowledge questionnaire was adapted from the validated survey of nutrition and health-related knowledge, attitudes, and practices related with nutrition during pregnancy and lactation developed by Food and Agriculture Organization of the United Nations [38] and UNICEF [39]. It included a section of six closed-answer questions with the possibility of more than one answer, and a second section with 29 questions about maternal and newborn nutrition was added (23 were true and 6 were false), with three options of answer: “True”, “False” and “I don’t know” [25,40]. For a score-based indicator of knowledge, each respondent was given a score based on the number of correct responses provided. Each correct answer was assigned one point, while an incorrect answer scored zero points; consequently, the level of nutritional knowledge was estimated as the mean percentage for correctly answered questions. If more than two-thirds (~67%) of physiotherapists correctly answered any of the questions, the score of the question was considered as high. The total score of the questionnaire can range from 0 (zero) to 35 points: a score between 0 and below 24 points was considered a reduced level of knowledge, and equal or above 24 points was considered a high level of knowledge. Participants who did not answer the question or reported incomplete information were excluded [38].

The survey was revised by a panel of two PhD holders in nutrition and in maternal and newborn health, one physiotherapist, and two experts in physiology in maternal health. A pre-test was carried out on a sample of 30 physiotherapists (results were not included in this study), and its validity was evaluated by the specialists with adequate internal consistency (Cronbach’s alpha of 0.88).

#### 2.2.3. Statistical Analysis

A statistical analysis was performed using SPSS software version 26.0 (SPSS, Inc., Chicago, IL, USA). The categorical variables are expressed as absolute counts and percentages and were compared using a chi-square test or Fisher’s exact test.

All quantitative variables were initially assessed for normality using the Shapiro–Wilk test, and an assessment of data asymmetry was performed through standard deviation. For comparison between two groups, either Student’s *t*-test or the Mann–Whitney test was used for normal and non-normal data, respectively. An independent samples *t*-test was applied to explore differences in nutritional knowledge scores between sex and professional experience-stratified variables, with Cohen’s d effect sizes interpreted as small (d = 0.20–0.49), medium (d = 0.50–0.79), and large (d = 0.80 and above). A regression analysis using an automatic linear regression model, aiming to improve model accuracy, was used to evaluate predictors of nutritional knowledge. Thus, regression standardized predicted values and residuals were computed iteratively, assuming linear models and adding variables considered significant by correlation analysis, using the forward stepwise multiple regression method. Statistical significance was established at *p* < 0.05.

## 3. Results

As shown in Table 1, from participating physiotherapists (32.2 ± 6.0 years old; 72.9% females and 27.1% males), 17.7% of females and 10.5% of males had a Master’s or PhD.

The majority had no specialization in maternal and child physiotherapy (96.1% of females and all males), only six female physiotherapists were professionally active in maternal and childcare (11.8%), and there were no male active physiotherapists in this area.

In addition, a small number of female physiotherapists (13.7%) reported having training in maternal and child nutrition during academic graduation, while a lower number was observed in the male group (5.3%). Participants’ professional experience was significantly different (*p* = 0.034), according to physiotherapist sex (Table 1).

Table 2 shows the nutritional knowledge percentages of physiotherapists regarding maternal and newborn health according to sex, which were calculated by cumulatively adding the correct responses to six knowledge questions. The mean score was 3.4 ± 1.0, ranging from 0 to 5 out of a possible score range from 0 to 6 points. Both female and male participants had the highest percentages in correct answers in the first two questions comparing the diet between a pregnant woman and a non-pregnant (73.4% and 26.6%) or a lactating woman and a non-breastfeeding woman (73.8% and 26.2%), respectively. It was also asked which supplements would benefit women during pregnancy, and the answers should have been both iron and folic acid supplements, but only 36.8% of female and 14.9% of male participants chose folic acid supplements, respectively (with a small effect size between participants’ sex, Cohen’s d = 0.305) (Table 2).

On the other hand, most female participants (58.3%) correctly considered that folic acid deficiency is a risk factor for fetal neural tube defects. In the question about the pregnant woman’s malnutrition and its risks to the newborn’s health, only 15.6% of the female physiotherapists and 9.1% of males considered maternal malnutrition as a factor for negative effects towards the baby’s growth and development and no male physiotherapists reported any concern about it (a small effect size between groups was observed, Cohen’s d = 0.443) (Table 2). The less correctly answered question was about the woman’s body needs for a period of two or three years between delivering until the next pregnancy, in which only 2.6% of female participants answered affirmatively, and all male participants answered incorrectly (Table 2).

Table 3 shows participant knowledge about maternal and child nutrition, according to sex. The mean score was 16.4 ± 3.1, ranging from 8 to 21 out of a possible score range of 0 to 29 points; a slight difference was observed between female and male physiotherapists (*p* < 0.05). The questions with the highest correct answers were numbers 4, 27, and 29 in female participants, and significant differences between female and male physiotherapists were observed in five questions (numbers 19, 21, 22, and 25; all with medium effect sizes, Cohen’s d = 0.50 and 0.79, exception for number 21) (Table 3). The first question presented a curious result as only one female participant chose the correct answer, and none of male professionals knew the answer. In question 2, there was no correct answer by either male or female participants. In question 12, only 8.6% of females and 2.9% of male physiotherapists answered correctly. It should be noted that questions 15, 17 and 23 obtained almost 100% incorrect answers, regardless of participant sex (Table 3). In addition, in more than half of questions, both sexes did not know how to answer, or did not report the correct answer.

Table 4 shows the physiotherapist nutritional knowledge percentages regarding maternal and newborn health, according to professional experience, calculated by cumulatively adding correct responses to six knowledge questions. The mean score was 3.4 ± 1.0, ranging from 0 to 5 out of a possible score range of 0 to 6 points. Both groups of professionals had the highest percentages in correct answers in the first two questions comparing diet between a pregnant woman and a non-pregnant (77.8% and 22.2%), or a lactating woman and a non-breastfeeding woman (74.8% and 25.2%), respectively. Regarding two types of supplements that women should benefit from during pregnancy, only 37.1% of the less experienced professionals and 11.2% of more experienced indicated iron and acid folic supplements as beneficial for most pregnant women (a small effect size between groups was observed, Cohen’s d = 0.239, *p* = 0.033) (Table 4). The importance of taking folic acid supplements during pregnancy was correctly answered by 58.3% of the less experienced and by 20.8% of more experienced professionals (a small effect size between groups was also observed, Cohen’s d = 0.377, *p* = 0.028) (Table 4).

Regarding a pregnant woman’s malnutrition and risks to the newborn’s health with low birthweight, only 8.2% of the more experienced physiotherapists answered correctly, which is of concern, as this question is related to the baby’s development and is within the physiotherapy field of action; furthermore, only 17.3% of the less experienced group correctly marked this option (Table 4).

Concerning the recommendation for a woman to wait from two to three years until the next pregnancy, only 4.8% of the less experienced group indicated that this recommendation is adequate to rebuild body stores; in addition, all more experienced health professionals answered incorrectly (Table 4).

Table 5 shows the physiotherapists’ knowledge about maternal and child nutrition according to professional experience. The mean score was 16.4 ± 3.1, ranging from 8 to 21 out of a possible score range of 0 to 29 points; also, a slight difference was observed between the less and more experienced physiotherapists (*p* < 0.05), as observed in female and male participants.

Significant differences between the less and more experienced physiotherapists were observed in question 21 (“Adequate water intake during pregnancy [including drinking water and other beverages, such as milk, natural juices and infusions, and foods rich in water—soups], salads and fruit is 2 L/day”) (Cohen’s d = 0.246, *p* < 0.05) (Table 5).

There were no correct answers for question 2 within both groups of participants, because pregnant women should avoid caffeine intake above 200 mg/day, not 400 mg/day, as mentioned in the question (Table 5). Also, the more experienced physiotherapists did not answer correctly to the question number 17 considering the moderated consumption of salt.

Overall, in more than half of the questions, both groups of physiotherapists did not know the answer or did not report the correct answer.

The comparison of total nutritional knowledge scores in relation to physiotherapist sex, professional experience, and educational level is shown in Table 6.

The total mean nutritional knowledge was higher in the female participants (20.3 ± 3.0) when compared with males (18.5 ± 3.7) (*p* = 0.041). Also, the total mean nutritional knowledge was higher in the more experienced physiotherapists (21.2 ± 2.2) when compared with less experienced physiotherapists (19.3 ± 3.5, *p* = 0.038) (Table 6).

A stepwise multiple regression analysis was applied to analyze the nutritional knowledge as the dependent variable and used professional experience and sex (female and male) as independent variables, as shown in Table 7. The model with professional experience and sex is statistically significant (F = 4.428, *p* < 0.001). The adjusted R-square value of 0.419 indicates that the 41.9% change in nutrition knowledge is explained by two variables (professional experience and sex). The coefficients of two significant variables in models (8.529 and 3.616) indicate that for the more experienced and female physiotherapists, the total nutrition knowledge scores increased by 8.529 units and by 3.616 units, respectively, when compared with the less experienced and male physiotherapists, respectively (*p* < 0.001 and *p* = 0.019, respectively).

## 4. Discussion

This study aimed to evaluate Brazilian physiotherapists’ nutritional knowledge about maternal and newborn health considering their sex and professional experience. Our findings are relevant as physiotherapists play a crucial role in supporting women during pregnancy and in postpartum, including their babies. They are also important as an adequate nutritional status is required to decrease the risk of persistent symptomatic diastasis recti abdominis [41] or pelvic floor dysfunction during delivery [27].

Also, our findings are consistent with other studies from Europe [42,43,44], the United States [45], Canada [46], Asia [47,48] and Africa [49], which have found that physicians’ nutritional knowledge is limited and insufficient compared with recommendations [42].

Furthermore, this theme has been poorly explored in the specific sample of physiotherapists regarding maternal and newborn health, contrarily to its importance for public health. To the best of our knowledge, this is the first study evaluating Brazilian physiotherapists’ nutritional knowledge about maternal and newborn health and well-being, as previous studies were mostly conducted in the general population attending public health services [50].

Overall, we found that most physiotherapists had poor nutritional knowledge, but the more experienced participants and females had better knowledge than less experienced and male physiotherapists, respectively. In addition, nutrition has not been much adopted in the academic curriculum of physiotherapy graduation courses in Brazil. A small number of female and male participants had the discipline of nutrition in their academic education because nutrition is not mandatory during the physiotherapy course. In fact, it was only present in some postgraduate and master’s courses. Only 13.7% of female physiotherapists had training in maternal and child nutrition during their graduation, and the number was even lower when compared to 5.3% of male participants. This may be why female physiotherapists demonstrated more nutritional knowledge in maternal and newborn health than their male counterparts, in addition to their willingness to accumulate expertise on pregnancy-related issues [15].

There was a lack of nutritional knowledge among these health professionals when considering the pregnant woman’s malnutrition and its risks to the newborn’s health with low-birthweight newborns and a woman’s needs to rebuild her body reserves of nutrients before a new pregnancy, which is in line with recent research [48]. Although the more experienced physiotherapists demonstrated more nutritional knowledge about maternal and newborn health than their less experienced counterparts, significant differences were only observed for the adequate amount of water intake during pregnancy. This may be why physiotherapists seek wider sources of knowledge in which they felt less prepared or trained. In a Taiwanese study, the competence of female physiotherapists was associated with their willingness to provide health education to women during pregnancy or in postpartum and with prior experience of treating antepartum or postpartum women [15].

Since pregnancy causes numerous bodily physiological changes, there is an increased need for essential nutrients, in particular folic acid and iron supplementation, during pregnancy [5,7]. Our results make clear the need for effective nutritional clarification to physiotherapist education regarding the effect of these supplements on maternal health and its biological interaction with the child’s growth and development, including the fetal neural tube. Folic acid supplements during pregnancy prevent birth defects/abnormalities in the fetal nervous system, as they are essential for the synthesis of deoxyribonucleic and ribonucleic acids, being fundamental in erythropoiesis, and totally indispensable in the regulation of the normal development of nerve cells, acting in the prevention of congenital defects in the fetal neural tube and megaloblastic anemia in the mother [51]. It is known that during pregnancy, there is a considerable increase in metabolic demands for iron because of the pregnant woman’s physiological expansion of hematopoiesis [5]. This increase is also justified by the fetus’ growth, the need for hemoglobin formation, and the baby’s central nervous system development through the production of enzymes responsible for brain metabolism [17]. It has been estimated that iron deficiency can directly compromise maternal and fetal health, since its deficiency is related to increased maternal mortality and morbidity, premature birth, and low-birth-weight newborns [5,7].

In questions related to breastfeeding and its associated benefits for the mother, female physiotherapists obtained more correct answers than males. In fact, breastfeeding can prevent breast, ovarian, and endometrial cancer, in addition to facilitating the loss of weight gained during pregnancy and preventing postpartum hemorrhages by facilitating the uterus’s faster return to its natural size [5]. Oxytocin is released at the time of childbirth, but its action is greater during breastfeeding, and because this hormone is responsible for uterine contractions, its release is reduced [51]. On the other hand, the relationship between breastfeeding and reduction in breast and ovarian epithelium cancer risk is associated with immune functions and the decreased function of neoplastic cells [52]. The benefits of breastfeeding are also very positive for newborn growth and development, operating effectively in nutritional, immunological, cognitive, social and economic aspects, acting as a preventive agent of diseases not only in childhood, but also in later life [53]. Also, the initial mother–infant relationship is especially important for both (mother and newborn). All our participants were unanimous about the nutritional characteristics of breast milk and its influence on the baby’s health.

Another finding worthy of attention from our study was that only two physiotherapists answered correctly for the question related with the recommended interval between pregnancies, as it is known that very short periods can increase the risk of premature or low-birth-weight newborns due to the mother’ s inability to recover her nutritional reserves between one pregnancy and another [54].

There is a consensus regarding the potential negative effects of a newborn’s low birthweight on their development that may be difficult to treat and reverse [53,54]. Therefore, a higher rate of correct answers on the matter was expected because this issue is a matter of the physiotherapist’ s responsibility. Therefore, this should be further discussed among these professionals to assess the origin of this lack of information. It is estimated that 10 to 15% of preterm infants develop a wide variety of neurological developmental sequelae during childhood, and that 30 to 40% have motor and behavioral changes, in addition to learning deficits at school age [55]. In addition, both prematurity and low birthweight are predisposing aspects for possible infections; higher hospitalization rates; developmental changes; postnatal neuropsychological deficits; lack of manipulative skills; delayed gait, coordination, and language skills; and poor future school performance [55]. Motor delay, when identified in advance, allows the child to be inserted into a specific program of skills work that will bring future benefits regarding the gain of certain movements and the child’s global development [56]. In fact, children who are born with low birthweight normally present developmental and motor sequelae and suffer from malnutrition during prenatal conditions [56]. Furthermore, the risk of changes in motor development increases proportionally as the newborn’s weight at birth decreases, and motor delay interfere with the child’s global development (cognition, language, self-care and socialization) [57]. In addition, the influence of the mother’s nutritional status on birth conditions emphasizes the importance of efficient prenatal nutritional monitoring to prevent gestational weight gain [5], which can directly influence the fetus’ growth, causing negative changes in newborn nutritional status and development [58].

Thus, our findings suggest that health professionals, including physiotherapists, should pay attention to inadequate gestational weight gain to avoid maternal and fetal health complications and future problems for the child’s psychomotor development [4].

Nutritional guidelines should be offered according to the economic, social, and cultural possibilities of each pregnant woman that imply the need for adequate education of physiotherapists. Physiotherapists play an important role in guiding and encouraging pregnant women towards a healthy and active lifestyle (including adequate nutritional habits) and identifying pregnant women at potential nutritional risk through a multidisciplinary team (together with nutritionists and medical doctors).

Overall, our results suggest that Brazilian universities offering bachelor’s degrees in physiotherapy need to include theoretical and practical components of nutrition in their curricula units. These components should be applied to maternal and newborn health to better educate students and prepare future health professionals. These courses may be offered in a limited number of Brazilian universities and do not reach a wide proportion of students because participation may be voluntary or optional. Therefore, the inclusion of nutrition (compulsory with, at least 120 h, during a full semester) within the academic curricula may be the best approach to obtain future physiotherapists with appropriate knowledge and skills to effectively assist pregnant women during pregnancy and in postpartum.

Our study was characterized by limitations. It was a cross-sectional study based on a small size sample, which may be insufficient to detect between-group differences in nutritional knowledge and attitudes; therefore, our findings may not represent the nutritional knowledge of Brazilian physiotherapists more broadly. Also, the use of an unvalidated instrument may not comprehensively assess the breadth of nutrition related to maternal and newborn health. Consequently, our results need to be further validated given that sample collection was highly affected by participants’ time-restrictions due to professional demands and COVID-19 restrictions, in addition to the lack of a specific instrument to assess the physiotherapists’ knowledge in this specific subject. Nevertheless, the joint work of specialists from different areas contributed to reaching a consensus instrument with a good internal consistency that was easy to understand by the respondents, i.e., without specialized training in nutrition.

## 5. Conclusions

Nutritional knowledge related to maternal and newborn health among Brazilian physiotherapists was found to be insufficient. There is a need to expand the education and knowledge of these health professionals regarding nutrition in association with maternal and newborn health, ensure better guidance for future pregnant women and children, and prevent risk factors associated with women’s health and lifestyle before, during, and after pregnancy. The integration of nutritional education and literacy regarding energy balance, nutritional requirements, and energy expenditure according to pregnant woman’s needs and physical activity level should be promoted as part of the curriculum of physiotherapy graduation and continuing education, as the subject has been rapidly developing. A comprehensive questionnaire examining physiotherapist nutritional knowledge and practice regarding maternal and newborn health in Brazil is required. Such research would also have a great biological impact on women and children’s health and development (specifically for vulnerable groups) and spur the improvement of healthcare services.

## Figures and Tables

**Table 1:** Age, education level, and professional experience of physiotherapists (51 females and 19 males).

Participants’ Characteristics	Females (*n* = 51)		Males (*n* = 19)		
	*Mean ± SD*	*Min.–Max.*	*Mean ± SD*	*Min.–Max.*	*p*
**Age** (years)	31.9 ± 6.2	20–49	33.0 ± 5.3	24–47	0.507
	***N***	***%***	***N***	***%***	***p***
** Education level in physiotherapy **					0.259
Graduation	51	100	19	100	
Post-graduation	9	17.7	2	10.5	
**Professional experience in physiotherapy**					0.034 *
≤10 years	37	72.5	15	78.9	
>10 years	14	27.4	4	21.0	
***Do you have any specialization in maternal and child physiotherapy?***					0.381
Yes	2	3.9	0	--	
No	49	96.1	19	100	
***If yes, which one?***					
Postgraduate studies	1	2.0	--	--	
Specialization course	1	2.0	--	--	
***Do you work professionally in the area of maternal and childcare?***					0.114
Yes	6	11.8	0	--	
No	45	88.2	19	100	
*** During your academic training, did you have any maternal and child nutrition discipline in the curriculum? ***					0.322
Yes	7	13.7	1	5.3	
No	44	86.3	18	94.7	

* *p*-value from chi-square test or *t*-test. Significance level: *p* < 0.05.

**Table 2:** Physiotherapists’ knowledge of nutrition in pregnancy, according to sex (51 females and 19 males).

Questions about the Physiotherapists’ Knowledge about Nutrition in Pregnancy	Responses	Correct Response(s)	Females (*n* = 51)		Males (*n* = 19)		Effect Size ^1^	*p*-Value ^2^
			N (%)	Mean ± SD	N (%)	Mean ± SD		
How should a pregnant woman eat relative to a non-pregnant woman to provide good nutrition to the baby and help it to grow?	184	Eating more food at each meal (eating more food per day) or eating more often (eating more times per day). Eating more protein-rich foods. Eating more iron-rich foods. Using iodized salt when preparing meals.	135 (73.4)	0.98 ± 0.14	49 (26.6)	0.84 ± 0.38	0.609	0.651
How should a lactating woman eat compared to a non-breastfeeding woman to be healthy and produce more breast milk?	141	Eating more food at each meal (eating more food per day) or eating more often (eating more times per day). Eating more protein-rich foods. Eating more iron-rich foods. Using iodized salt when preparing meals.	104 (73.8)	0.78 ± 0.42	37 (26.2)	0.79 ± 0.42	0.012	0.983
Most women would benefit from two types of supplements, or pills, during pregnancy. Which are they?	141	Iron supplements. Folic acid supplements.	52 (36.8)	0.86 ± 0.35	21 (14.9)	0.79 ± 0.42	0.199	0.035 *
Why is it so important to take folic acid supplements during pregnancy?	72	To prevent birth defects/abnormalities in the fetal nervous system (brain, spine and skull).	42 (58.3)	0.35 ± 0.48	15 (20.8)	0.21 ± 0.42	0.305	0.971
When a pregnant woman is undernourished, she is at risk of having a low-birth-weight baby, which means the baby is small or has a low birth weight. What are the risk factors for the health of these babies?	186	Slower growth and development. Risk of being undernourished/deficient in micronutrients. Others.	29 (15.6)	0.41 ± 0.50	17 (9.1)	0.63 ± 0.50	0.443	0.001 **
It is recommended that a woman wait at least two or three years between pregnancies before becoming pregnant again. Why is there such a recommendation?	76	To rebuild your body stores of nutrients (fat, iron and others)	2 (2.6)	0.04 ± 0.20	0	0 ± 0	0.233	--
Mean of the knowledge score (Mean ± SD) (Minimum–Maximum)		3.4 ± 1.0 (0–5)	3.4 ± 0.9 (1–5)		3.3 ± 1.2 (0–5)		0.168	0.534

^1^ Effect sizes reported for the Cohen’s d test. ^2^ *p*-values reported for independent samples *t*-test. * Significant differences for *p* < 0.05. ** Significant differences for *p* < 0.01.

**Table 3:** Physiotherapist nutritional knowledge about maternal and child nutrition, according to sex (51 females and 19 males).

Physiotherapists’ Nutritional Knowledge	Responses	Correct Response	Females (*n* = 51)		Males (*n* = 19)		Effect Size ^1^	*p*-Value ^2^
			N (%)	Mean ± SD	N (%)	Mean ± SD		
1. Eat 5 to 6 meals a day, more or less every 3 h: breakfast (breakfast), lunch and dinner and 2 to 3 small snacks.	70	True	1 (1.4)	0.04 ± 0.20	0	--	0.233	0.296
2. Pregnant women should avoid caffeine intake above 400 mg/day.	70	False	0	--	0	--	--	--
3. Prioritize the consumption of vegetables, starting meals with a vegetable soup.	70	True	23 (32.9)	0.47 ± 0.50	7 (10.0)	0.37 ± 0.50	0.204	0.752
4. Breast milk is the most complete and adequate food for the healthy growth and development of the baby.	69	True	49 (71.0)	0.96 ± 0.20	18 (26.1)	0.95 ± 0.23	0.65	0.552
5. The pregnant woman should eat “for two”, due to the increase in her energy needs.	70	False	40 (57.1)	0.78 ± 0.42	17 (24.3)	0.89 ± 0.32	0.282	0.950
6. Prefer fatty fish (salmon, herring, tuna, sardines) and white meats, such as poultry and rabbit.	70	True	32 (45.7)	0.63 ± 0.49	12 (17.1)	0.63 ± 0.50	0.008	0.899
7. Limit red meat consumption to 2 or 3 times a week.	70	True	19 (27.1)	0.37 ± 0.49	10 (14.3)	0.53 ± 0.51	0.311	0.613
8. Consume about half of cereals, such as bread, rice and pasta, in wholegrain form.	70	True	29 (41.4)	0.57 ± 0.50	6 (8.6)	0.32 ± 0.48	0.512	0.143
9. Eat 3 to 4 servings of fruit a day.	70	True	46 (65.7)	0.90 ± 0.30	16 (22.9)	0.84 ± 0.38	0.186	0.311
10. Unsanitized raw vegetables and fruits can be consumed with care.	70	False	43 (61.4)	0.84 ± 0.37	16 (22.9)	0.84 ± 0.38	0.003	0.560
11. Breast milk reduces the incidence of allergies, respiratory infections and ear infections.	70	True	45 (64.3)	0.88 ± 0.33	19 (27.1)	1.00 ± 0.00	0.422	0.147
12. A woman with a Body Mass Index between 18.5–24.9 kg/m^2^ can increase her body weight by 7.0–11.5 kg.	70	False	6 (8.6)	0.12 ± 0.33	2 (2.9)	0.11 ± 0.32	0.038	0.249
13. Eat 3 servings of low-fat or low-fat dairy a day.	70	True	19 (27.1)	0.37 ± 0.49	3 (4.3)	0.16 ± 0.38	0.466	0.209
14. Meat, fish and undercooked eggs should be avoided.	70	True	37 (52.9)	0.73 ± 0.45	17 (24.3)	0.89 ± 0.32	0.404	0.184
15. Breastfeeding facilitates the loss of the weight gained by women during pregnancy and helps the uterus recover its normal size more quickly, also favouring the prevention of postpartum hemorrhages.	70	False	1 (1.4)	0.90 ± 0.30	1 (1.4)	0.63 ± 0.50	0.746	0.989
16. Always prefer vegetable oils, such as olive oil and lard.	70	True	28 (40.0)	0.55 ± 0.50	5 (7.1)	0.26 ± 0.45	0.584	0.191
17. Moderate salt consumption, using little salt for cooking, not adding salt to the dish and avoiding products and foods with excess salt.	70	True	1 (1.4)	0.92 ± 0.27	1 (1.4)	0.89 ± 0.32	0.095	0.877
18. A woman with a Body Mass Index between 25.0–29.9 kg/m^2^ can increase her body weight by 11.5–16.0 kg.	70	False	20 (28.6)	0.39 ± 0.49	3 (4.3)	0.16 ± 0.38	0.504	0.806
19. Breastfeeding reduces the risk of developing osteoporosis, breast, ovarian and endometrial (uterus) cancer.	70	True	35 (50.0)	0.69 ± 0.47	7 (10.0)	0.37 ± 0.50	0.668	0.007 **
20. Unpasteurized milk or dairy products should be avoided.	70	True	32 (45.7)	0.63 ± 0.49	15 (21.4)	0.79 ± 0.42	0.344	0.163
21. Adequate water intake during pregnancy [including drinking water and other beverages (such as milk, natural juices and infusions) and foods rich in water (soups, salads and fruit)] is 2 L/day.	70	False	5 (7.1)	0.10 ± 0.30	3 (4.3)	0.16 ± 0.38	0.186	0.027 *
22. The best sources of calcium are milk, dairy products and some cereals.	70	True	35 (50.0)	0.69 ± 0.47	6 (8.6)	0.32 ± 0.48	0.787	0.020 *
23. Preconception, pregnant or breastfeeding women should receive a daily Iodine supplement in the form of potassium iodide—150 to 200 μg/day.	70	True	4 (5.7)	0.08 ± 0.27	2 (2.9)	0.11 ± 0.32	0.095	0.335
24. Usually, folic acid supplementation starts 3 months before conception and continues through the first six months of pregnancy.	70	False	2 (2.9)	0.04 ± 0.20	2 (2.9)	0.11 ± 0.32	0.283	0.116
25. Iron is important for energy metabolism and for the development of the fetal nervous system.	70	True	45 (64.3)	0.88 ± 0.33	12 (17.1)	0.63 ± 0.50	0.663	0.006 **
26. To improve iron absorption, pregnant women should avoid drinking tea or coffee with main meals (consume 1–2 h before or after).	70	True	29 (41.4)	0.57 ± 0.50	8 (11.4)	0.42 ± 0.51	0.294	0.222
27. Vitamin D is essential for the fixation of calcium and is essential for the formation of the baby’s skeleton and teeth.	70	True	48 (68.6)	0.94 ± 0.24	18 (26.1)	0.95 ± 0.23	0.026	0.922
28. Breast milk strengthens the child’s immune system and prevents diarrhoea.	70	True	43 (61.4)	0.84 ± 0.37	18 (26.1)	0.95 ± 0.23	0.310	0.312
29. Folic acid plays a key role in reducing the risk of developing a baby’s neural tube malformations.	70	True	49 (70.0)	0.96 ± 0.20	18 (26.1)	0.95 ± 0.23	0.065	0.809
Mean of the knowledge score (Mean ± SD) (Minimum–Maximum)	16.4 ± 3.1 (8–21)		16.8 ± 3.0 (8–21)		15.2 ± 3.0 (10–21)		0.541	0.043 *

^1^ Effect sizes reported for Cohen’s d test. ^2^ *p*-values reported for independent samples *t*-test. * Significant differences for *p* < 0.05. ** Significant differences for *p* < 0.01.

**Table 4:** Physiotherapists’ knowledge of nutrition in pregnancy, according to professional experience (52 physiotherapists with 10 years or less of experience and 18 physiotherapists with more than 10 years of experience).

Questions about the Physiotherapists’ Knowledge about Nutrition in Pregnancy	Responses	Correct Response(s)	≤10 Years (*n* = 52)		>10 Years (*n* = 18)		Effect Size ^1^	*p*-Value ^2^
			N (%)	Mean ± SD	N (%)	Mean ± SD		
How should a pregnant woman eat relative to a non-pregnant woman to provide good nutrition to the baby and help it to grow?	185	Eating more food at each meal (eating more food per day) or eating more often (eating more times per day). Eating more protein-rich foods. Eating more iron-rich foods. Using iodized salt when preparing meals.	144 (77.8)	0.94 ± 0.24	41 (22.2)	0.94 ± 0.24	0.009	0.001 **
How should a lactating woman eat compared to a non-breastfeeding woman to be healthy and produce more breast milk?	143	Eating more food at each meal (eating more food per day) or eating more often (eating more times per day). Eating more protein-rich foods. Eating more iron-rich foods. Using iodized salt when preparing meals.	107 (74.8)	0.77 ± 0.43	36 (25.2)	0.83 ± 0.38	0.154	0.02 *
Most women would benefit from two types of supplements, or pills, during pregnancy. Which are they?	143	Iron supplements. Folic acid supplements.	53 (37.1)	0.87 ± 0.35	16 (11.2)	0.78 ± 0.43	0.239	0.033 *
Why is it so important to take folic acid supplements during pregnancy?	72	To prevent birth defects/abnormalities in the fetal nervous system (brain, spine and skull).	42 (58.3)	0.27 ± 0.45	15 (20.8)	0.44 ± 0.51	0.377	0.028 *
When a pregnant woman is undernourished, she is at risk of having a low-birth-weight baby, which means the baby is small or has a low birth weight. What are the risk factors for the health of these babies?	110	Slower growth and development. Risk of being undernourished/deficient in micronutrients. Others.	19 (17.3)	0.50 ± 0.51	9 (8.2)	0.39 ± 0.50	0.220	0.084
It is recommended that a woman wait at least two or three years between pregnancies before becoming pregnant again. Why is there such a recommendation?	42	To rebuild your body stores of nutrients (fat, iron and others)	2 (4.8)	0.04 ± 0.19	0	0.00 ± 0.00	0.229	--
Mean of the knowledge score (Mean ± SD) (Minimum–Maximum)	3.4 ± 1.0 (0–5)		3.4 ± 1.0 (0–5)		3.4 ± 0.9 (2–5)		0.004	0.988

^1^ Effect sizes reported for Cohen’s d test. ^2^ *p*-values reported for independent samples *t*-test. * Significant differences for *p* < 0.05. ** Significant differences for *p* < 0.01.

**Table 5:** Physiotherapists’ nutritional knowledge about maternal and child nutrition, according to professional experience (52 physiotherapists with 10 years or less of experience and 18 physiotherapists with more than 10 years of experience).

Physiotherapists’ Nutritional Knowledge	Responses	Correct Response	≤10 Years (*n* = 52)		>10 Years (*n* = 18)		Effect Size ^1^	*p*-Value ^2^
			N (%)	Mean ± SD	N (%)	Mean ± SD		
1. Eat 5 to 6 meals a day, more or less every 3 h: breakfast (breakfast), lunch and dinner and 2 to 3 small snacks.	70	True	48 (68.6)	0.04 ± 0.19	18 (25.7)	0.0 ± 0.0	0.229	0.06
2. Pregnant women should avoid caffeine intake above 400 mg/day.	70	False	0	--	0	--	--	--
3. Prioritize the consumption of vegetables, starting meals with a vegetable soup.	70	True	22 (31.4)	0.44 ± 0.50	8 (11.4)	0.44 ± 0.51	0.004	0.501
4. Breast milk is the most complete and adequate food for the healthy growth and development of the baby.	69	True	52 (74.3)	0.94 ± 0.24	18 (25.7)	1.00 ± 0.00	0.283	0.227
5. The pregnant woman should eat “for two”, due to the increase in her energy needs.	70	False	39 (55.7)	0.75 ± 0.44	18 (25.7)	1.00 ± 0.00	0.660	0.294
6. Prefer fatty fish (salmon, herring, tuna, sardines) and white meats, such as poultry and rabbit.	70	True	32 (45.7)	0.62 ± 0.49	12 (17.1)	0.67 ± 0.49	0.105	0.748
7. Limit red meat consumption to 2 or 3 times a week.	70	False	11 (15.7)	0.37 ± 0.49	3 (4.2)	0.56 ± 0.51	0.386	0.094
8. Consume about half of cereals, such as bread, rice and pasta, in wholegrain form.	70	True	23 (32.9)	0.44 ± 0.50	12 (17.1)	0.67 ± 0.49	0.451	0.299
9. Eat 3 to 4 servings of fruit a day.	70	True	46 (65.7)	0.88 ± 0.32	16 (22.9)	0.89 ± 0.32	0.013	0.41
10. Unsanitized raw vegetables and fruits can be consumed with care.	70	False	44 (62.9)	0.81 ± 0.40	17 (24.3)	0.94 ± 0.24	0.375	0.514
11. Breast milk reduces the incidence of allergies, respiratory infections and ear infections.	70	True	47 (67.1)	0.90 ± 0.30	17 (24.3)	0.94 ± 0.24	0.143	0.295
12. A woman with a Body Mass Index between 18.5–24.9 kg/m^2^ can increase her body weight by 7.0–11.5 kg.	70	False	6 (8.6)	0.12 ± 0.32	2 (2.9)	0.11 ± 0.32	0.013	0.934
13. Eat 3 servings of low-fat or low-fat dairy a day.	70	True	15 (21.4)	0.27 ± 0.45	8 (11.4)	0.44 ± 0.51	0.377	0.325
14. Meat, fish and undercooked eggs should be avoided.	70	True	39 (55.7)	0.75 ± 0.44	15 (21.4)	0.83 ± 0.38	0.196	0.237
15. Breastfeeding facilitates the loss of the weight gained by women during pregnancy and helps the uterus recover its normal size more quickly, also favouring the prevention of postpartum hemorrhages.	70	False	1 (1.4)	0.85 ± 0.36	1 (1.4)	0.78 ± 0.43	0.179	1
16. Always prefer vegetable oils, such as olive oil and lard.	70	True	25 (35.7)	0.48 ± 0.51	8 (11.4)	0.44 ± 0.51	0.072	0.314
17. Moderate salt consumption, using little salt for cooking, not adding salt to the dish and avoiding products and foods with excess salt.	70	True	2 (2.9)	0.88 ± 0.32	0	1.00 ± 0.00	0.413	--
18. A woman with a Body Mass Index between 25.0–29.9 kg/m^2^ can increase her body weight by 11.5–16.0 kg.	70	False	16 (22.9)	0.31 ± 0.47	7 (10.0)	0.39 ± 0.50	0.171	0.185
19. Breastfeeding reduces the risk of developing osteoporosis, breast, ovarian and endometrial (uterus) cancer.	70	True	30 (42.9)	0.58 ± 0.50	12 (17.1)	0.67 ± 0.49	0.181	0.104
20. Unpasteurized milk or dairy products should be avoided.	70	True	32 (45.7)	0.62 ± 0.49	15 (21.4)	0.83 ± 0.38	0.467	0.062
21. Adequate water intake during pregnancy [including drinking water and other beverages (such as milk, natural juices and infusions) and foods rich in water (soups, salads and fruit)] is 2 L/day.	70	False	8 (11.4)	0.13 ± 0.35	1 (1.4)	0.06 ± 0.24	0.246	0.04 *
22. The best sources of calcium are milk, dairy products and some cereals.	70	True	30 (42.9)	0.56 ± 0.50	12 (17.1)	0.67 ± 0.49	0.219	0.717
23. Preconception, pregnant or breastfeeding women should receive a daily Iodine supplement in the form of potassium iodide—150 to 200 μg/day.	70	True	5 (7.1)	0.10 ± 0.30	1 (1.4)	0.06 ± 0.24	0.143	0.232
24. Usually, folic acid supplementation starts 3 months before conception and continues through the first six months of pregnancy.	70	False	3 (4.3)	0.06 ± 0.24	1 (1.4)	0.06 ± 0.24	0.009	0.095
25. Iron is important for energy metabolism and for the development of the fetal nervous system.	70	True	43 (61.4)	0.81 ± 0.40	15 (21.4)	0.83 ± 0.38	0.065	0.055
26. To improve iron absorption, pregnant women should avoid drinking tea or coffee with main meals (consume 1–2 h before or after).	70	True	26 (37.1)	0.48 ± 0.51	12 (17.1)	0.67 ± 0.49	0.372	0.068
27. Vitamin D is essential for the fixation of calcium and is essential for the formation of the baby’s skeleton and teeth.	70	True	49 (70.0)	0.94 ± 0.24	17 (24.3)	0.94 ± 0.24	0.009	0.079
28. Breast milk strengthens the child’s immune system and prevents diarrhoea.	68	True	45 (66.2)	0.87 ± 0.35	16 (23.5)	0.89 ± 0.32	0.069	0.086
29. Folic acid plays a key role in reducing the risk of developing a baby’s neural tube malformations.	70	True	49 (70.0)	0.94 ± 0.24	18 (25.7)	1.00 ± 0.00	0.283	0.073
Mean of the knowledge score (Mean ± SD) (Minimum–Maximum)	16.4 ± 3.1 (8–21)		17.8 ± 2.1 (8–21)		15.9 ± 3.3 (8–21)		0.619	0.027 *

^1^ Effect sizes reported for Cohen’s d test. ^2^ *p*-values reported for independent samples *t*-test. * Significant differences for *p* < 0.05.

**Table 6:** Association between the total nutrition score and the main study variables.

Variables	Total Nutrition Knowledge Score		*p*-Value ^1^
	Mean ± SD	Min.–Max.	
Total (*n* = 70)	19.8 ± 3.3	11–25	
Sex			
Female (*n* = 51)	20.3 ± 3.0	13–25	0.041 *
Male (*n* = 19)	18.5 ± 3.7	11–25	
Professional experience			
≤10 years (*n* = 52)	19.3 ± 3.5	11–25	0.038 *
>10 years (*n* = 18)	21.2 ± 2.2	16–24	
Educational level			
Bachelor’s (*n* = 57)	19.7 ± 3.2	13–25	0.466
Master’s (*n* = 6)	22.3 ± 2.0	20–25	
Ph.D. (*n* = 5)	19.6 ± 3.4	16–23	

SD: standard deviation. ^1^ A *t*-test was used to compare the means of two groups, and the F-test was used to compare variances of three or more groups. * Significant differences for *p* < 0.05.

**Table 7:** Stepwise multiple regression analysis between nutritional knowledge and study variables.

Model	Unstandardized Coefficients				95.0% Confidence Interval for B		
	B	Std. Error	*t*-Value	*p*-Value	Lower Bound	Upper Bound	
1 (Constant)	21.507	1.715	12.539	<0.001	18.083	24.930	R = 0.631 Adjusted R^2^ = 0.389 MSE = 4.686 F-test = 4.319
Professional experience	8.552	1.285	6.657	<0.001	5.988	11.116	
2 (Constant)	14.723	3.632	4.054	<0.001	7.471	21.974	R = 0.660 Adjusted R^2^ = 0.419 MSE = 4.570 F-test = 4.428
Professional experience ^1^	8.529	1.253	6.806	<0.001	6.027	11.030	
Sex ^2^	3.616	1.719	2.104	0.019	0.185	7.048	

^1^ Professional experience ≤ 10 years and >10 years; ^2^ Sex = male and female.

## Data Availability

The authors confirm that the data supporting the findings of this study are available within the article.

## References

[1] Wawrzyniak A., Pietruszka B. (2023). Dietary Habits and Nutritional Status of Different Population Groups in Relation to Lifestyle Factors and Nutritional Knowledge. Nutrients.

[2] Guiné R.P.F., Florença S.G., Aparício G., Cardoso A.P., Ferreira M. (2023). Food Literacy Scale: Validation through Exploratory and Confirmatory Factor Analysis in a Sample of Portuguese University Students. Nutrients.

[3] Morris J., Nikolopoulos H., Berry T., Jain V., Vallis M., Piccinini-Vallis H., Bell R.C., ENRICH team (2017). Healthcare providers’ gestational weight gain counselling practises and the influence of knowledge and attitudes: A cross-sectional mixed methods study. BMJ Open.

[4] Gilmore L.A., Klempel-Donchenko M., Redman L.M. (2015). Pregnancy as a window to future health: Excessive gestational weight gain and obesity. Semin. Perinatol..

[5] Silva M.-R.G., Doñate B.R., Santos-Rocha R. (2022). Nutritional and energy requirements of the pregnant exerciser and athlete. Exercise and Sporting Activity during Pregnancy.

[6] Rasmussen K.M., Yaktine A., Institute of Medicine (2009). Weight gain during pregnancy. Reexamining the Guidelines.

[7] Silva M.-R.G., Bellotto M.L. (2015). Nutritional Requirements for Maternal and Newborn Health. Curr. Women’s Health Rev..

[8] Institute of Medicine (2013). Implementing Guidelines on Weight Gain and Pregnancy.

[9] Silva M.-R.G., Paiva T., Santos-Rocha R., Simões V., Pimenta N. (2016). Neurophysiology, associated diseases to sleep deprivation and sleep hygiene in children. Active School—Physical Activity and Health in School Context.

[10] Franco V.F., Rodrigues A.S., Junior E., de Godói L.G., Monroy N., da Costa R.A., Francisco R. (2022). Demographic and epidemiological characteristics of pregnant and postpartum women who died from severe acute respiratory syndrome in Brazil: A retrospective cohort study comparing COVID-19 and nonspecific etiologic causes. PLoS ONE.

[11] Silva M.-R.G., Silva H.-H., Paiva T. (2018). Sleep duration, body composition, dietary profile and eating behaviours among children and adolescents: A comparison between Portuguese acrobatic gymnasts. Eur. J. Pediatr..

[12] Nogueira H., Costeira E., Pereira M.M., Costa D., Gama A., Machado-Rodrigues A., Silva M.R., Marques V.R., Padez C.M. (2020). The environment contribution to gender differences in childhood obesity and organized sports engagement. Am. J. Human. Biol..

[13] Branco M., Santos Rocha R., Vieira F., Silva M.-R.G., Aguiar L., Veloso A. (2016). Influence of body composition on gait kinetics throughout pregnancy and postpartum period. Scientifica.

[14] Perumal N., Wang D., Darling A.M., Liu E., Wang M., Ahmed T., Christian P., Dewey K.G., Kac G., Kennedy S.H. (2023). Suboptimal gestational weight gain and neonatal outcomes in low and middle income countries: Individual participant data meta-analysis. BMJ Clin. Res..

[15] Lin K.Y., Tsai Y.J., Yang J.F., Wu M.H. (2022). Physical therapists’ experiences and perceptions of antepartum and postpartum care. BMC Pregnancy Childbirth.

[16] Toloza F., Motahari H., Maraka S. (2020). Consequences of Severe Iodine Deficiency in Pregnancy: Evidence in Humans. Front. Endocrinol..

[17] Sousa A.F., Claro M.L., Rondó P. (2021). Screening for neuropsychomotor and social-emotional development in children under 24 months of age in the Brazilian semi-arid region. Rev. Paul. Pediatr..

[18] De Maeyer E.M. (1989). Preventing and Controlling Iron Deficiency through Primary Care.

[19] Multiple Micronutrient Supplements in Pregnancy: Implementation Considerations for Successful Integration into Existing Programmes. https://www.who.int/news-room/events/detail/2015/08/18/default-calendar/meetingmultiple-micronutrient-supplements-in-pregnancy.

[20] Santos-Rocha R., Szumilewicz A., Wegrzyk J., Hyvärinen M., Silva M.-R.G., Jorge R., Oviedo-Caro M.A. (2023). Active Pregnancy Guide—Physical Activity, Nutrition, and Sleep (e-Book).

[21] O’Connor D.L., Blake J., Bell R., Bowen A., Callum J., Fenton S., Gray-Donald K., Rossiter M., Adamo K., Nutrition Working Group (2016). Canadian consensus on female nutrition: Adolescence, reproduction, menopause, and beyond. J. Obstet. Gynaecol. Can..

[22] Australian Government (2015). Healthy Eating When You’re Pregnant or Breastfeeding.

[23] American College of Obstetricians and Gynecologists (2015). Nutrition during Pregnancy.

[24] Swedish National Food Agency (2015). Food for You Who Are Pregnant.

[25] Teixeira D., Pestana D., Calhau C., Vicente L., Graça P. (2015). Food and Nutrition in Pregnancy.

[26] Torró-Ferrero G., Fernández-Rego F.J., Jiménez-Liria M.R., Agüera-Arenas J.J., Piñero-Peñalver J., Sánchez-Joya M., Fernández-Berenguer M.J., Rodríguez-Pérez M., Gomez-Conesa A. (2022). Effect of physical therapy on bone remodelling in preterm infants: A multicenter randomized controlled clinical trial. BMC Pediatr..

[27] Zhu H., Zhang D., Gao L., Liu H., Di Y., Xie B., Jiao W., Sun X. (2022). Effect of Pelvic Floor Workout on Pelvic Floor Muscle Function Recovery of Postpartum Women: Protocol for a Randomized Controlled Trial. Int. J. Environ. Res. Public Health.

[28] Grieger J.A., Clifton V.L. (2014). A review of the impact of dietary intakes in human pregnancy on infant birthweight. Nutrients.

[29] Trumbo P., Schlicker S., Yates A.A., Poos M., Food and Nutrition Board of the Institute of Medicine, The National Academies (2022). Dietary reference intakes for energy, carbohydrate, fiber, fat, fatty acids, cholesterol, protein and amino acids. J. Am. Diet. Assoc..

[30] Food and Nutrition Board/Institute of Mdicine (2001). Dietary Reference Intakes for Vitamin A, Vitamin K, Arsenic, Boron, Chromium, Copper, Iodine, Iron, Manganese, Molybdenum, Nickel, Silicon, Vanadium, and Zinc.

[31] Food and Nutrition Board/Institute of Medicine (1999). Dietary Reference Intakes for Thiamin, Riboflavin, Niacin, Vitamin B6, Folate, Vitamin B12, Pantothenic Acid, Biotin, and Choline.

[32] Food and Nutrition Board/Institute of Medicine (2000). Dietary Reference Intakes for Vitamin C, Vitamin E, Selenium, and Carotenoids.

[33] Food and Nutrition Board/Institute of Medicine (2011). Dietary Reference Intakes for Calcium and Vitamin D.

[34] Food and Nutrition Board/Institute of Medicine (2005). Dietary Reference Intakes for Water, Potassium, Sodium, Chloride, and Sulfate.

[35] Elias M.C., Gomes D.L., Paracampo C.C.P. (2022). Associations between Orthorexia Nervosa, Body Self-Image, Nutritional Beliefs, and Behavioral Rigidity. Nutrients.

[36] Dean A.G., Sullivan K.M., Soe M.M. OpenEpi: Open Source Epidemiologic Statistics for Public Health, Version. www.OpenEpi.com.

[37] Brazilian General Council for Physiotherapy and Occupational Therapy—4th Region of Minas Gerais Statistics. https://estatisticas.app.appery.io/app/ScreenPrincipal.html.

[38] Macías Y.F., Glasauer P. (2014). KAP Manual: Guidelines for Assessing Nutrition-Related Knowledge, Attitudes and Practices.

[39] UNICEF Demographic and Health Surveys. https://data.unicef.org/.

[40] Candeias V. (2010). Breastfeeding.

[41] Olsson A., Kiwanuka O., Wilhelmsson S., Sandblom G., Stackelberg O. (2019). Cohort study of the effect of surgical repair of symptomatic diastasis recti abdominis on abdominal trunk function and quality of life. BJS Open.

[42] Han S.L., Auer R., Cornuz J., Marques-Vidal P. (2016). Clinical nutrition in primary care: An evaluation of resident physicians’ attitudes and self-perceived proficiency. Clin. Nutr. ESPEN.

[43] Mowe M., Bosaeus I., Rasmussen H.H., Kondrup J., Unosson M., Rothenberg E., Irtun Ø., Group S.N. (2008). Insufficient nutritional knowledge among health care workers?. Clin. Nutr..

[44] Ozcelik A., Surucuoglu M., Akan L. (2007). Survey on the Nutrition Knowledge Level of Turkish Physicians: Ankara as a Sample. Pak. J. Nutr..

[45] Vetter M.L., Herring S.J., Sood M., Shah N.R., Kalet A.L. (2008). What do resident physicians know about nutrition? An evaluation of attitudes, self-perceived proficiency and knowledge. J. Am. Coll. Nutr..

[46] Temple N.J. (1999). Survey of nutrition knowledge of Canadian physicians. J. Am. Coll. Nutr..

[47] Hu S.P., Wu M.Y., Liu J.F. (1997). Nutrition knowledge, attitude and practice among primary care physicians in Taiwan. J. Am. Coll. Nutr..

[48] Kraemer K., Beesabathuni K., Askari S., Khondker R., Khan T.U., Rahman M., Gibson S., Merritt R., Bajoria M., Lingala S. (2023). Knowledge, Attitudes and Practices of Pregnant Women and Healthcare Providers in Bangladesh regarding Multivitamin Supplements during Pregnancy. Healthcare.

[49] Alkhaldy A.A. (2019). Nutritional Knowledge and Self-Reported Nutritional Practice against Malnutrition among Physicians in Jeddah, Saudi Arabia. Healthcare.

[50] Gonçalves I., Pereira P.F., Silva M., Ladeira F.B., Moreira T.R., Cotta R., da Costa G.D. (2020). Nutritional status coverage trend registered in the SISVAN web in seven municipalities of the Zona Da Mata Mineira, Brazil, from 2008 to 2017, and its association with socio-economic, demographic and organisation of health system variables. J. Nutr. Sci..

[51] Anelli G.M., Parisi F., Sarno L., Fornaciari O., Carlea A., Coco C., Porta M.D., Mollo N., Villa P.M., Guida M. (2022). Associations between Maternal Dietary Patterns, Biomarkers and Delivery Outcomes in Healthy Singleton Pregnancies: Multicenter Italian GIFt Study. Nutrients.

[52] Antunes M.B., Rossi R.M., Pelloso S.M. (2020). Relationship between gestational risk and type of delivery in high risk pregnancy. Rev. Esc. Enferm. USP.

[53] Cavalcanti S.H., Caminha M., Figueiroa J.N., Serva V.M., Cruz R., de Lira P.I., Batista Filho M. (2015). Factors associated with breastfeeding practice for at least six months in the state of Pernambuco, Brazil. Braz. J. Epidemiol..

[54] Lima R.M., Leite E.V.N.C., Furtado D.F., Santos A.M. (2020). Prevalence and factors associated with the consumption of folic acid and iron in pregnant women in the BRISA cohort. Rev. Bras. Saude Matern. Infant..

[55] Ribeiro C.C., Pachelli M.R.O., Amaral N.C.O., Lamônica D.A.C. (2017). Development skills of children born premature with low and very low birth weight. CoDAS.

[56] Perkins J.M., Kim R., Krishna A., McGovern M., Aguayo V.M., Subramanian S.V. (2017). Understanding the association between stunting and child development in low- and middle-income countries: Next steps for research and intervention. Soc. Sci. Med..

[57] Li S.J., Tsao P.N., Tu Y.K., Hsieh W.S., Yao N.J., Wu Y.T., Jeng S.F. (2022). Cognitive and motor development in preterm children from 6 to 36 months of age: Trajectories, risk factors and predictability. Early Hum. Dev..

[58] Øverby N.C., Hillesund E.R., Sagedal L.R., Vistad I., Bere E. (2015). The Fit for Delivery study: Rationale for the recommendations and test-retest reliability of a dietary score measuring adherence to 10 specific recommendations for prevention of excessive weight gain during pregnancy. Matern. Child. Nutr..

